# Biologically synthesized of Au/Pt/ZnO nanoparticles using *Arctium lappa* extract and cytotoxic activity against leukemia

**DOI:** 10.1007/s10544-020-00526-z

**Published:** 2020-10-10

**Authors:** Renata Dobrucka, Aleksandra Romaniuk-Drapała, Mariusz Kaczmarek

**Affiliations:** 1 Department of Non-Food Products Quality and Packaging Development, Institute of Quality Science, Poznań University of Economics and Business, al. Niepodległości 10, 61-875 Poznań, Poland; 2 Department of Clinical Chemistry and Molecular Diagnostics, Poznan University of Medical Sciences, 49 Przybyszewskiego St, 60-355 Poznań, Poland; 3 Department of Cancer Immunology, Chair of Medical Biotechnology, Poznan University of Medical Sciences, Garbary 15 Str, 61-866 Poznan, Poland; 4 Gene Therapy Laboratory, Department of Cancer Diagnostics and Immunology, Greater Poland Cancer Centre, Garbary 15 Str, 61-866 Poznan, Poland

**Keywords:** Biological synthesis, Au/Pt/ZnO nanoparticles, *Arctium lappa* extract

## Abstract

The main objective of this work was to assess the cytotoxic activity of Au/Pt/ZnO nanoparticles synthesized using *Arctium lappa* extract against leukemia. The Au/Pt/ZnO nanoparticles obtained as a result of biological synthesis were characterized by UV-Vis, Scanning (SEM) and Transmission electron microscopy (TEM), Fourier transform infrared spectroscopy (FTIR), and Atomic Force Microscopy (AFM). The applied methods showed that the size of nanoparticles ranged from 10 to 40 nm. This work also assessed the cytotoxicity of Au/Pt/ZnO nanoparticles by means of MTT assay, and analyzed apoptosis as well as the influence of the cultivation time and concentration of Au/Pt/ZnO nanoparticles on the percentage of dead cells. The studies showed that the percentage of dead leukemia cells increased with the cultivation time and concentration of Au/Pt/ZnO nanoparticles. There was observed an increase in the percentage of cells in the G2/M phase, which suggests the stoppage of G2/M leading to cell death. The cytotoxicity of Au/Pt/ZnO nanoparticles determined by means of the MTT test indicated that the viability of leukemia cells practically disappeared when the concentration of the tested nanoparticles was 10 mol.

## Introduction

Metal nanoparticles obtained from nanotechnology have gained global interest because of their broad applicability in biomedical and physiochemical fields. Monometallic nanoparticles are used for many different purposes (Nascimento et al. 2018). Biomedical application present nanoporous materials which take important role for drug delivery (Li et al. 2018a; Li et al. 2018b). Also, the control over the porous structures is fundamental to elucidate the relationship between the structures and material performance (Li et al. 2019a; Jiang et al. 2018). In the case of metal nanoparticles the interesting solutions are derived from the combination of metal nanoparticles. When combined, they tend to demonstrate new or stronger characteristics, different from the ones exhibited by their individual components (Zhang et al. 2010). It is beneficial to prepare multimetallic nanoparticles of various sizes and shapes, as they are better than their mono- and bimetallic counterparts in terms of the degree of selectivity, activity and chemical/physical stability (Basavegowda et al. 2017). Trimetallic nanoparticles have recently gained the attention of scientists. Thanks to the synergy between their respective components, trimetallic nanoparticles exhibit new properties, both chemical and physical ones, and for this reason they are causing a greater research awareness.

The world literature depicts various physical and chemical methods for producing trimetallic nanoparticles. However, the majority of those methods are expensive and create a potential risk to the environment and organisms. In this decade, the focus on obtaining eco-friendly products in eco-friendly ways is increasing, and we are trying to find alternative solutions (Hasan et al. 2018). One example of that is the biological synthesis of metal nanoparticles. The eco-friendly production of nanoparticles is possible with the use of biological organisms – such as microorganisms, plant extract or plant biomass – as a replacement for chemical and physical methods. Biological methods utilize plant extracts because they are available, safe to handle, and rich in metabolites. In addition, plant extracts act as reducing and capping agents in the process of nanoparticle synthesis. Therefore, recently much attention has been directed to synthetic approaches that are not harmful to the environment. Unfortunately, there are few examples of the biological synthesis of trimetallic nanoparticles in the literature.

For this reason, in this work, we obtained Au/Pt/ZnO nanoparticles from *A. lappa* extract. *A.lappa* L. is a perennial plant commonly known as burdock. It is a popular edible plant used in traditional medicine worldwide. It belongs to the family of *Asteraceae*, one of the largest families of flowering plants. It is native to Europe and Asia, and the early European settlers rapidly spread it across North America. Traditionally, *A. lappa* was used in order to treat various infections, including sore throat, boils, rashes and skin disorders (Don & Yap 2019). *A.lappa* flowers appear on the top of large, hard and fleshy stems which grow from the rosette. The most commonly used part of *A. lappa* is the root, which is thick and fleshy and can be even 50 cm long. Extracts from different parts of *A. lappa* L. have been reported to possess different biological properties; they exhibit, among others, anti-oxidant, anti-cancer, anti-diabetic, anti-tubercular, anti-inflammatory, anti-bacterial and anti-viral activity. Studies have confirmed that *A. lappa* L. contains bioactive molecules of various kinds, including flavonoids, polyphenols, oligosaccharides, and polyunsaturated fatty acids (Li et al. 2019b). In this work, due to the characteristics of biologically active compounds, Au/Pt/ZnO nanoparticles were obtained from *A. lappa* extract, and then they were assessed in terms of their effects on leukemia. The obtained nanoparticles were tested against leukemia because this blood cancer is characterized by the widespread uncontrolled proliferation of a large number of abnormal white blood cells, which invade the bone marrow and often spill out into the blood stream (Soni & Yadav 2015).

## Materials and methods

### Synthesis of au/Pt/ZnO nanoparticles

The synthesis of Au/Pt/ZnO nanoparticles started with the preparation of the extract. The extract was prepared by combining 10 g of powdered *A. lappa* and 100 ml of double distilled water. The prepared solution was subjected to heating and vigorous stirring for 65 min at 80 °C. Then, the extract was filtered through Whatman’s No. 1 filter paper, and used immediately. Then, the following solutions were prepared: 5 mM HAuCl_4_, 5 mM K_2_PtCl_6_ and 5 mM ZnNO_3_. The solutions were combined with the extract at a 1:1 ratio. The solution made this way was stirred at 75 °C for 24 h, and the UV-absorption spectrum of Au/Pt/ZnO nanoparticles was monitored after 12 and 24 h.

### Characterization of au/Pt/ZnO nanoparticles

In order to measure the maximum absorbance of the sample, UV-Visible spectrophotometry was used. The optical properties of Au/Pt/ZnO nanoparticles biosynthesized using *A. lappa* extract were analyzed by means of ultraviolet and visible absorption spectroscopy (spectrophotometer Cary E 500) in the range of 300 nm–600 nm. The binding properties of Au/Pt/ZnO nanoparticles biosynthesized using *A. lappa* extract were examined by means of FTIR analysis. The Au/Pt/ZnO nanoparticles biosynthesized using *A. lappa* extract were characterized by means of Fourier transform infrared spectroscopy (FTIR). A Transmission electron microscope JEOL JEM 1200 EXII, operating at 80 kV, was used to determine the shape, size and microstructures of Au/Pt/ZnO nanoparticles biosynthesized using *A. lappa* extract. The study used the atomic force microscope INTEGRA SPECTRA SOLAR of NT-MDT brand and measurement tips dedicated for NSGO1 high-resolution measurements, and it was carried out in the tapping mode. The resonance frequency of the tips was from 87 to 230 kHz. The force constant was from 1.45 to 15.1 N/m. The scanning area was 10 μm × 10 μm. There were 1000 × 1000 scanning points within the scanning area. Scanning electron microscopy (SU3500), Hitachi, was used to obtain the picture of Au/Pt/ZnO nanoparticles biosynthesized using *A. lappa* extract

### Evaluation of cytotoxic activity of au/Pt/ZnO nanoparticles

In this work, the influence of Au/Pt/ZnO nanoparticles on the vitality of human cells was evaluated in vitro, with the use of established human Acute T Cell Leukemia cell line, Jurkat (ATCC® TIB-152™), as well as mononuclear cells isolated from peripheral blood (PBMC) of voluntary donors. PBMC contains both peripheral blood lymphocytes (PBL) and monocytes. Cells were cultured in suspension in RPMI 1640 medium with 2 mM L-glutamine with the addition of 10% fetal bovine serum (FBS) and 1% Gibco® Antibiotic-Antimycotic solution (10,000 units/mL of penicillin, 10,000 μg/mL of streptomycin, and 25 μg/mL of Amphotericin B) on 24-well plastic plates (TC-PLATE 24 well, Greiner). Cell cultures were carried out in an incubator at 37 °C in a 5% CO_2_ atmosphere with increased humidity. The Au/Pt/ZnO nanoparticles have been added in three various concentrations, 1 μM, 10 μM, and 100 μM. As controls served the samples without the Au/Pt/ZnO nanoparticles addition. All tested samples, both Jurkat cells, and PBMC were cultured for 24, 48 and 72 h.

### PBMC separation

Whole peripheral blood samples from voluntary donors were collected in heparin tubes. Then blood was mixed in a 1:1 ratio with sterile Phosphate-Buffered Saline (PBS) and dropped on the top layer of Histopaque®-1077 solution (Sigma-Aldrich). Histopaque®-1077 is a solution of polysucrose and sodium diatrizoate with a density of 1.077 g/mL which allows the easy separation of viable lymphocytes and other mononuclear cells from small volumes of whole blood. After 20 min of centrifugation at 1500 rpm in RT, the buffy coats layer with mononuclear cells were aspirated and plated on the culture 24-well plates.

### MTT test (cell viability assay)

Cell survival and the index of the half-maximal inhibitory concentration (IC50) were analyzed with the MTT test. The level of cytotoxicity was determined by the estimation of the percentage of dead cells and the degree of their inhibition of growth. During the MTT test is measured the reduction of water-soluble MTT tetrazolium salts (3-(4,5-dimethylthiazol-2-yl)-2,5-diphenyltetrazolium bromide) to blue-violet insoluble formazan crystals by active mitochondrial dehydrogenase. This assay allows for the quantitative evaluation of cell proliferation based on the measurement of the linear relationship between cell activity and absorbance of the color reaction. Prior to the reading absorbance, the formazan crystals were extracted from the cells and solubilized with 10% SDS in 0.01 M HCl solution. The absorbances were measured by using a microplate reader (Multiscan, Labsystems, Thermo Fisher Scientific Inc.) at 570/690 nm wavelengths.

Jurkat cells were plated to the culture wells in the amount of 5 × 10^3^ cells/well. Next, cells cultured with the addition of Au/Pt/ZnO nanoparticles in conditions described above were treated with 10 μl of MTT solution (5 mg/ml thiazolyl blue Tetrazolium Bromide). Followed by 4 h incubation the formazan crystals were released from the cells with 100 μl of a solubilizing solution. Test results were given as Relative Viability of Cells (RVC), which is defined as the ratio between the absorbance value for cells cultured with the presence of nanoparticles tested, to absorbance value of control samples. The RVC value was calculated from the formula described below, were (a) denotes the absorbance of the tested sample; (b) the absorbance of the blank control (pure medium without the cells) and (c) the absorbance of the control cells without addition Au/Pt/ZnO nanoparticles.

$$ \mathrm{RVC}\left(\%\right)=\left[\left(\mathrm{a}-\mathrm{b}\right)/\left(\mathrm{c}-\mathrm{b}\right)\right]\times 100 $$

Moreover, the IC50 index was calculated. IC50 value indicates the concentration of tested substances needed for inhibition, in vitro, of biological activity of cells by 50%.

### Apoptosis evaluation with Annexin V

Assessment of the impact of Au/Pt/ZnO nanoparticles on the viability of the Jurkat cell line was also carried out in terms of the initiation of apoptosis or necrosis. Evaluation of apoptotic or necrotic death was performed using a commercially available FITC Annexin V Apoptosis Detection Kit I (BD Pharmingen), in accordance with the manufacturer’s protocol. Analogously to the MTT assessment, cells were cultured in the presence of 1 μM, 10 μM, and 100 μM of Au/Pt/ZnO nanoparticles during 24, 48, and 72 h. The assay was performed as follows, at the beginning cells were suspended in 100 μl of Annexin buffer (1×) in amounts of 1 × 10^5^ cells. Next, to the samples 5 μl of propidium iodide (PI) and 5 μl of Annexin V conjugated with fluorescein (FITC) were added. After 15 min of incubation in the dark, 400 μl of Annexin buffer (1×) was added to each test tube. Stained samples were acquired with FACS Canto flow cytometer (Becton Dickinson) and results were analyzed with use FACS Diva Software (Becton Dickinson). Cells were defined as early or late apoptotic or necrotic depending on the proportion between FITC and/or PI fluorescence.

### Evaluation of cell cycle

The proliferative activity of the Jurkat cell line and PBMC cells cultured in the presence of Au/Pt/ZnO nanoparticles was evaluated on the basis of the cell cycle. For this was used fluorescent dye intercalating into DNA structure, propidium iodide (PI). In this assay, the percentage of cells is determined on the basis of the mean fluorescence intensity (MFI) emitted by PI which intercalates into the DNA of replicating cells. MFI emitted by PI is depending on the phase of the cell cycle. The measure of proliferative activity is the percentage of cells in the S phase of the cell cycle. The number of cells in the S phase of the cell cycle allows establishing the level of cells in the replication process. Additionally, based on cytometric histograms is possible to determine the percentage of cells prior to the mitosis (G2/M phase) and the percentage of dead cells.

For the assay cells were transferred onto culture plates in triplicates in amount 5 × 10^4^ cells per well and cultured in the presence of Au/Pt/ZnO nanoparticles in concentrations 1 μM, 10 μM, and 100 μM through 24, 48, and 72 h. Next, cells were prepared as follow, firstly cells were permeabilized using BD Perm/Wash™ Buffer (BD Biosciences) and next stained with a 100 μg/ml PI solution (Sigma-Aldrich). Both incubations were performed for 30 min in the fridge at 4 °C protected from light. Finally, stained cells were added to the acquisition with the use of the FACS Canto flow cytometer (Becton Dickinson). Analyses of histograms were performed using FACS Diva software (Becton Dickinson).

## Results and discussion

### UV VIS studies of au/Pt/ZnO nanoparticles

**Fig. 1:**
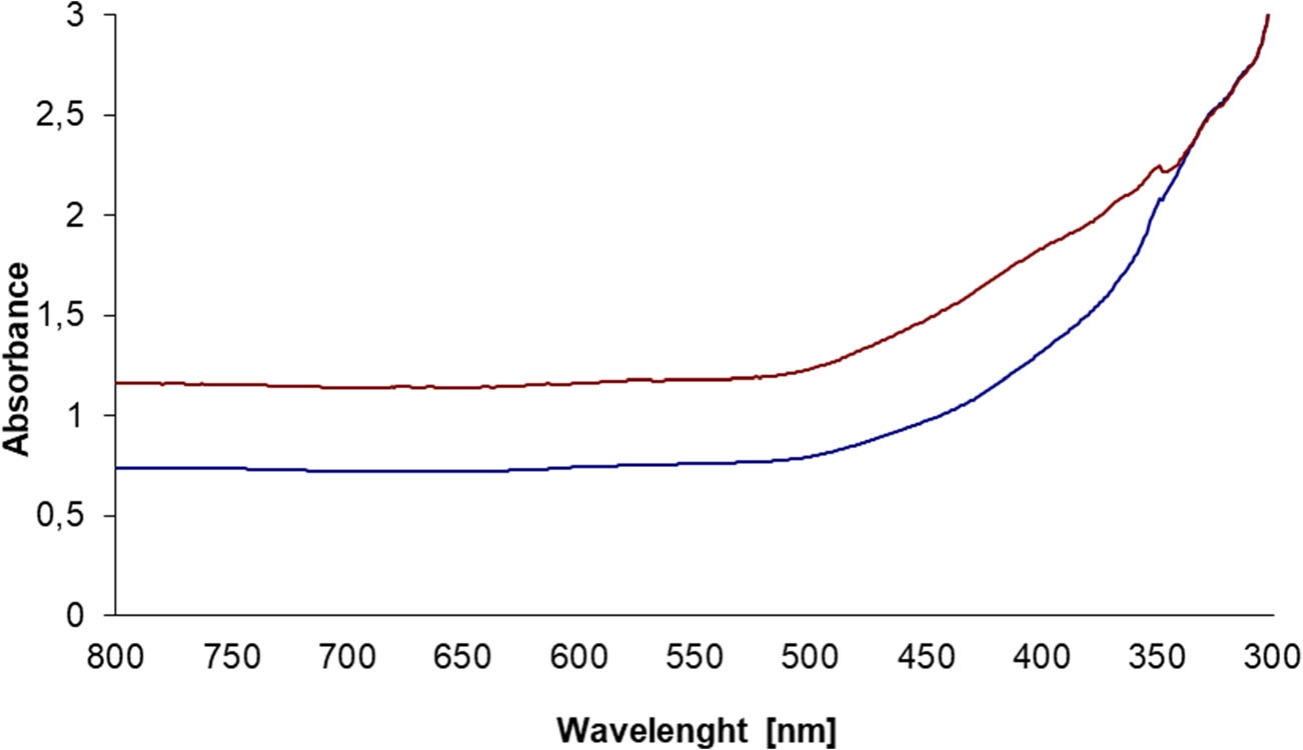
UV–visible spectra of Au/Pt/ZnO nanoparticles biosynthesized using of using *A. lappa* extract

The formation and stability of Au/Pt/ZnO nanoparticles were observed by means of UV–Vis spectrophotometry. Figure 1 presents the UV–visible spectra of Au/Pt/ZnO nanoparticles biosynthesized using *A.lappa* extract. The UV-absorption spectrum of the Au/Pt/ZnO nanoparticles biosynthesized using *A.lappa* extract was monitored after continuous stirring for 12 h and 24 h of reaction at 75 °C. When the extract was mixed with metal nanoparticle precursors, the solution rapidly changed its color from light beige to dark beige. Such a clear change of color confirms the reduction of metals to the nano form. Absorbance increased over reaction time. As regards the reaction media, their absorption spectra showed absorbance at 580 nm, confirming the presence of gold nanoparticles. There was also observed an intense peak at the wave length of 350 nm, which is characteristic of ZnO nanoparticles. It is confirmed, among others, by the studies carried out by Vijayakumar et al., (Vijayakumar et al. 2016), in which the absorption spectra of ZnO nanoparticles were observed between 330 nm and 370 nm.

### Fourier transform infrared spectroscopy (FTIR) studies of au/Pt/ZnO nanoparticles

**Fig. 2:**
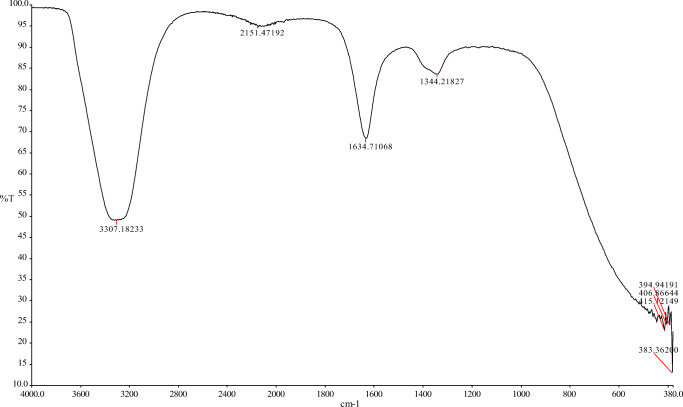
FTIR spectra of Au/Pt/ZnO nanoparticles biosynthesized using of using *A.lappa extract*

The FT-IR measurement was conducted in the wave number range from 380 to 4000 cm − 1 with the use of the KBr method, at room temperature. The interpretation of the IR spectrum includes the correlation between the absorption bands (vibrational bands) and the chemical compounds present in the sample. It is also possible to identify the plant extract biomolecules responsible for the processes of reduction and stabilization of the synthesized Au/Pt/ZnO nanoparticles (Senthilkumar & Sivakumar 2014). The strong peaks presented in Fig. 2 were observed at 3307 cm − 1, 2151 cm − 1, 1634 cm − 1, 1344 cm − 1, 415 cm − 1, 406 cm − 1, 394 cm − 1 and 383 cm − 1. The strong absorption peak at 3307 cm − 1 is related to -OH stretching and the aliphatic methylene group -C-H stretching. The peak at 2151 cm-1 may indicate the alkynes group. The most intense band at 1634 cm − 1 is related to amide bonds of proteins that may occur due to carboxyl stretching. The absorption band at 1344 cm-1 corresponds to C-H bending vibrations of the aromatic tertiary amine group. The absorption bands at 415 cm − 1, 406 cm − 1, 394 cm − 1 and 383 cm − 1 indicated the creation of metal-biomolecules found in the extract. The comparison of the presence of the OH group and the C–O group of acid in the extracts ascertains their role in the process of salt reduction into respective nanoparticles (Nadeem et al. 2019).

Based on the literature, the root of *A. lappa* is rich in polyacetylenes (trideca-1,11-dien-3,5,7,9-tetrain), arctinol, arctinal, arctinon, guaiane lactones (lappaphen), lignans (arctiin (1%), matairesinol, lappaol), flavonoids, phenolic acids 2–3.5% (caffeic acid, chlorogenic acid), guanidino-n-butyrate, inulin (30–45%), phytosterols (stigmasterol, beta-sitosterol, (campesterol), essential oil 0.1–0.2% (benzaldehyde, phenylacetaldehyde), potassium, magnesium and calcium salts, mucilage, sesquiterpene bitter (arctiopicrin) (Różański 2007; Liu et al. 2014).

Phenolic acids present in the roots of *A. lappa*, for example caffeoylquinic acid derivatives and chlorogenic acid, exhibit strong antioxidant activity, which is caused by the structure of phenolic acids (Jiang et al. 2016). In numerous studies on the antioxidant properties of phenolic acids, scientists have shown that such properties depend on chemical structure, i.e. they are related to the number of hydroxyl groups in the particle and the degree of their esterification. In compounds with one hydroxyl group, the antioxidant activity is additionally increased by the presence of one or two methoxy groups in the ring. The introduction of a group with electron donors, i.e. an alkyl group or a methoxy group, in the *ortho–* position strengthens the stability of the antioxidant properties of phenolic acids (Parus 2013). Additionally, flavonoids such as luteolin and quercetin, have free-radical scavenging activity and anti-inflammatory activity (Don & Yap 2019). Due to the increasing evidence of functional polysaccharides’ contributions to a variety of health beneficial properties, polysaccharides in *A. lappa* L. have received increasing scientific interest (Liu et al. 2014). Polysaccharides are the major constituents in the roots of *A. lappa* L., and they are used as an inulin source. Therefore, most published literatures focused on structure identification and bioactivities of fructan. Monosaccharide compositions and ratios influenced the antioxidant activity of polysaccharides. Meng et al. demonstrated that radical-scavenging activity was significantly correlated with mannose and glucose contents but not with galactose content. The studies conducted by Jiang et al. (Jiang et al. 2019) showed that polysaccharides from *A. lappa L*. could be used as natural antioxidants.

### Atomic force microscopy (AFM) studies of au/Pt/ZnO nanoparticles

**Fig. 3:**
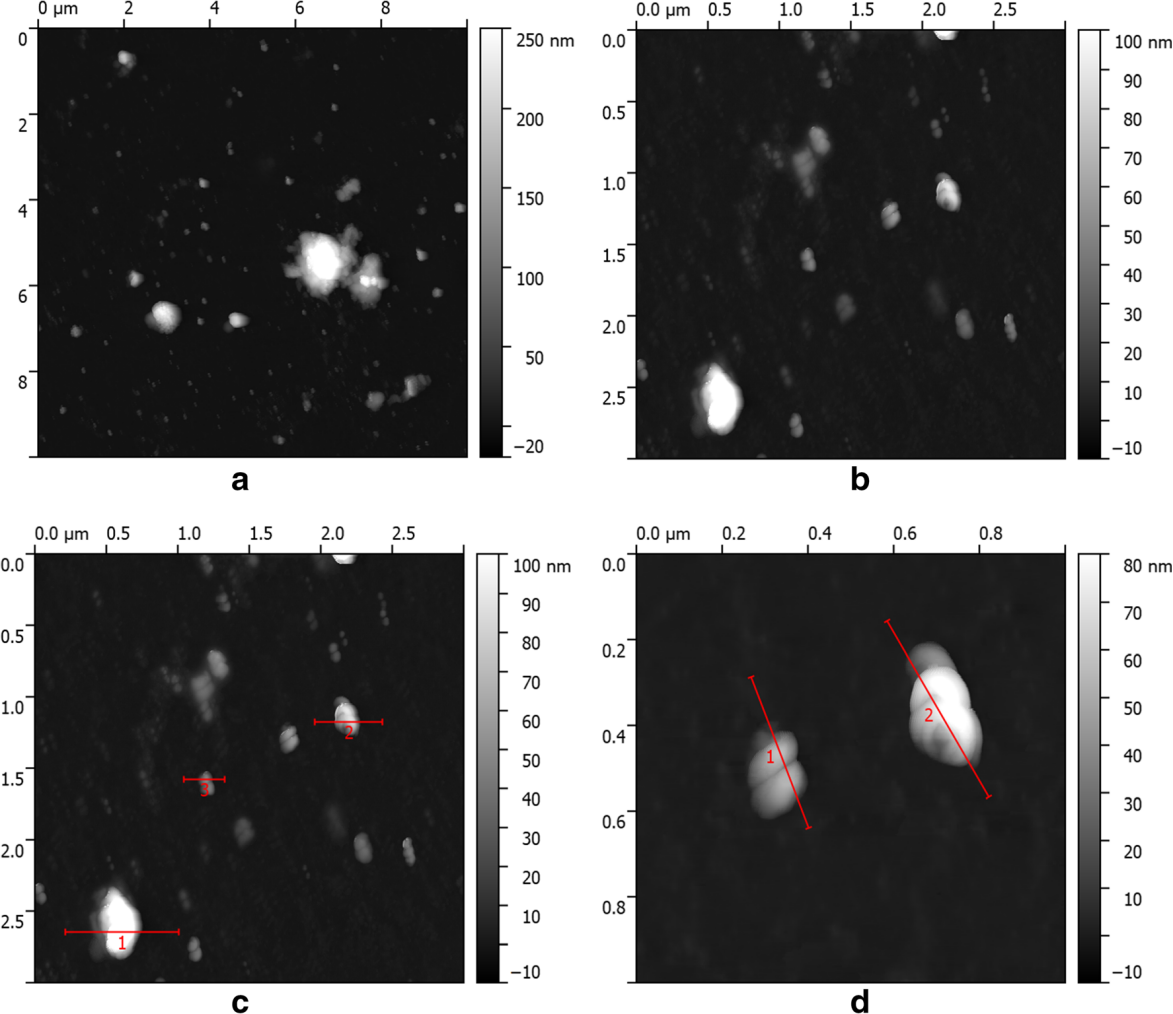
AFM image of Au/Pt/ZnO nanoparticles biosynthesized using of using *A. lappa* extract

The Au/Pt/ZnO nanoparticles biosynthesized using *A. lappa* extract were measured by means of Atomic Force Microscopy (AFM). AFM microscopes can be used to carry out topographic measurements and to determine some mechanical properties of the samples, such as elasticity, adhesion force and friction. Figure 3 presents the AFM image of Au/Pt/ZnO nanoparticles biosynthesized using *A. lappa* extract with: (A) the topography 10 μm × 10 μm, (B) the topography 3 μm × 3 μm, (C) the topography 3 μm × 3 μm with the profile and (D) the topography 1 μm × 1 μm with the profile. Based on topography, there were observed single particles of 10–40 nm and single agglomerates of particles of about 60 nm.

### Transmission Electron microscope analysis (TEM) and scanning electron microscopy (SEM) studies of au/Pt/ZnO nanoparticles

**Fig. 4:**
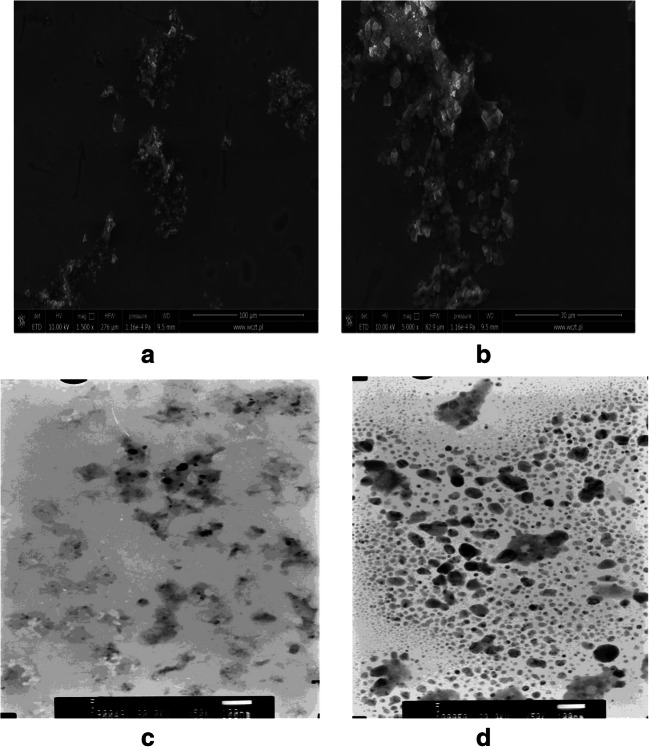
SEM and TEM image Au/Pt/ZnO nanoparticles biosynthesized using of using *A. lappa* extract

The size and shape of the obtained nanoparticles were analyzed by means of Scanning (SEM) and Transmission Electron Microscopy (TEM). The size of the particles was from 10 to 40 nm, and they were mostly spherical in shape. The findings were confirmed by transmission electron microscopy (TEM). Agglomerates of about 60 nm were observed as well. This analysis confirmed the previous Atomic Force Microscopy (AFM) measurements. Figure 4 shows the SEM images with the scale bar of (A) 100 μm and (B) 30 μm; and TEM images with the scale bar of (C) 100 nm and (D) 200 nm.

### Cytotoxic activity of au/Pt/ZnO nanoparticles

#### MTT assay

**Fig. 5:**
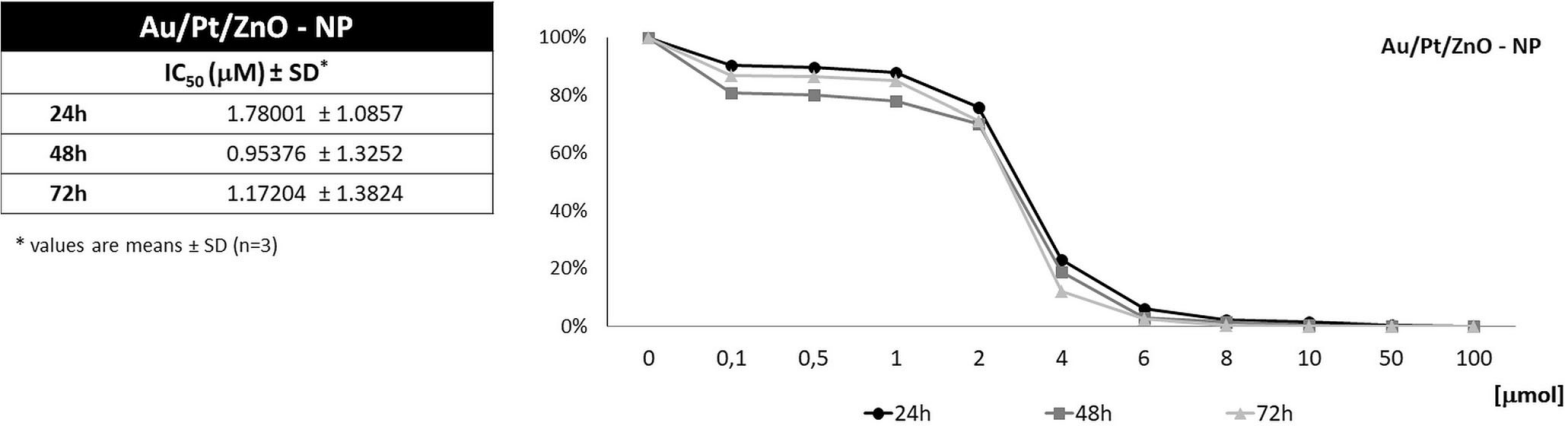
The Relative Cell Viability curve based on the MTT test to Jurkat cell line cultured through 24, 48 and 72 h in the presence of various concentrations of Au/Pt/ZnO nanoparticles

The cytotoxicity of the studied Au/Pt/ZnO nanoparticles was determined by MTT assay after culturing of Jurkat cell line in ten model concentrations: 0.1 μmol; 0.5 μmol; 1 μmol; 2 μmol; 4 μmol; 6 μmol; 8 μmol; 10 μmol; 50 μmol, and 100 μmol after 24, 48 and 72 h. Control cells were cultured without the presence of Au/Pt/ZnO nanoparticles. The values of Relative Cell Viability were significantly reduced at concentrations of nanoparticles from 1 μmol to 4 μmol. In this concentration range, the observed decrease was between 80% and 20%. Cell viability practically disappeared at a concentration of 10 μmol of tested nanoparticles. The IC_50_ index ranged from 0.95 μmol to 1.78 μmol for various cell culture times (Fig. 5).

#### Apoptosis assessment

**Fig. 6:**
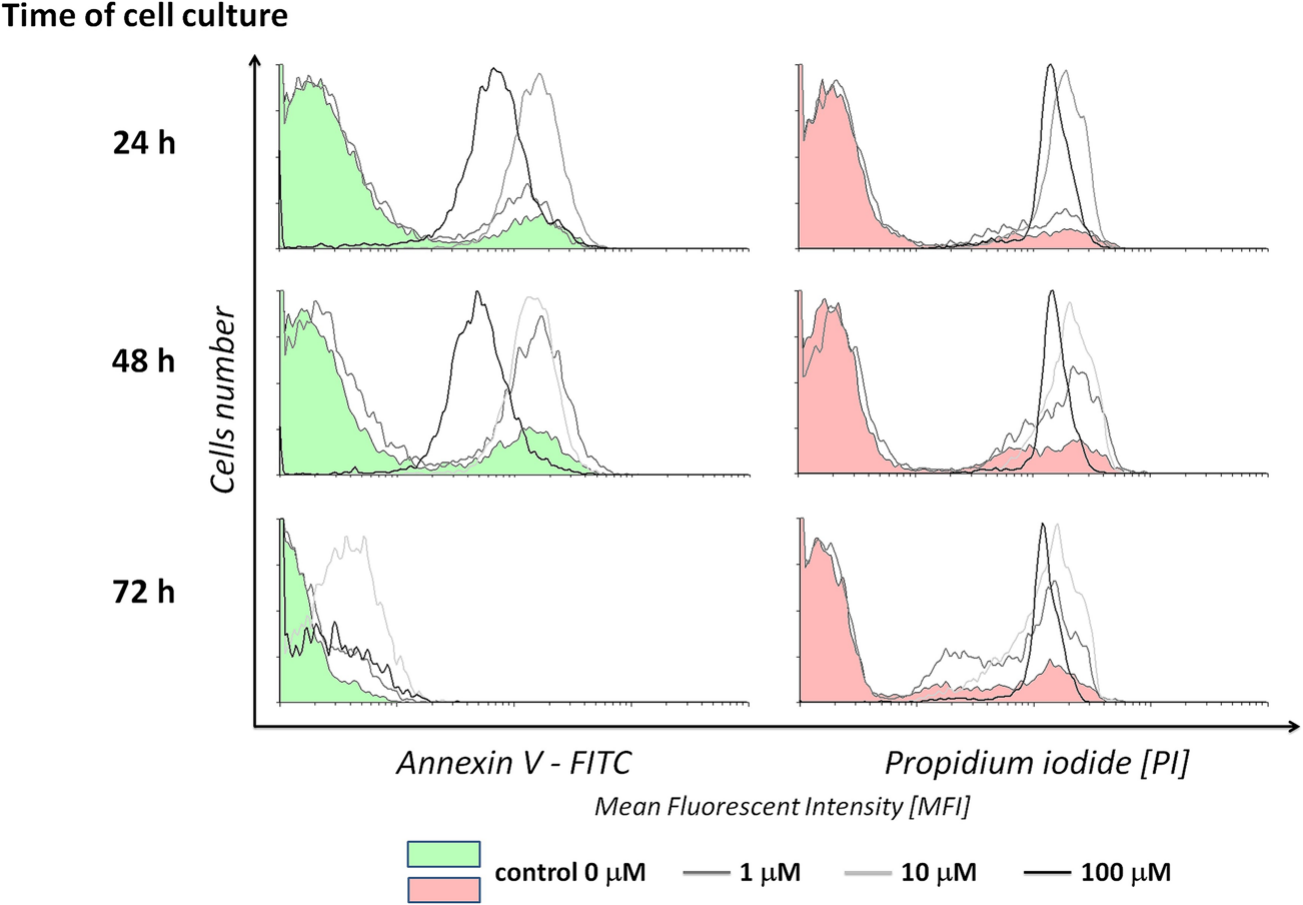
Mean Fluorescence Intensity of fluorescein-bonded Annexin V (FITC) and propidium iodide (PI) used for staining of apoptotic and necrotic Jurkat cells cultured through 24, 48, and 72 h in various concentrations of Au/Pt/ZnO nanoparticles

Apoptosis is a process of programmed cell death that occurs in multicellular organisms, and is considered the preferred way to eliminate tumor cells (Liang et al. 2019). To determine the type of cell death of Jurkat cell lines cultured in the presence of Au/Pt/ZnO nanoparticles a functional test based on FITC-labeled Annexin V and propidium iodide was used (Fig. 6).

**Fig. 7:**
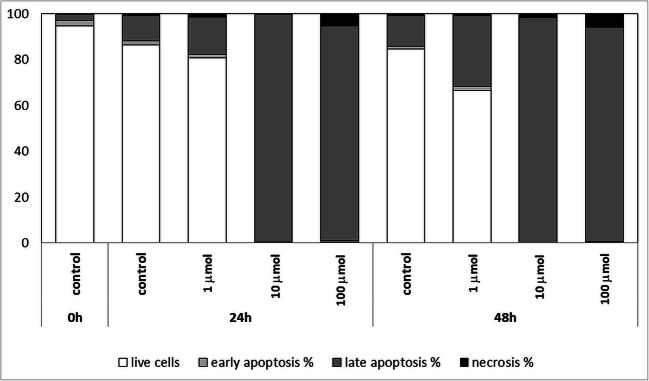
Percentages of Jurkat cells cultured for 24, and 48 h in the presence of different concentrations of Au/Pt/ZnO nanoparticles showing a state of early apoptosis, late apoptosis and necrosis

For cancer cells, uncontrolled cell growth and differentiation along with loss of apoptotic functions leading to a massive expansion in neoplastic cell population causes cancer progression (Abotaleb et al. 2018; Igney & Krammer 2002). In our studies, cells were tested at a concentration of 1 μmol, 10 μmol and 100 μmol for 24 and 48 h. The tested Au/Pt/ZnO nanoparticles were found to cause the death of Jurkat cells. The type of death and its intensity depended on the concentration of the tested nanoparticles and the time of cell exposure. The Au/Pt/ZnO nanoparticles quickly induced apoptosis of over 90% of cells already in 24 h of culture at a concentration of 10 μmol. This result confirmed the previous MTT test results. Concentrations of 10 μmol and 100 μmol practically killed all studied cells. After 48 h of culture, even nanoparticles used at a concentration of 1 μmol stimulated cellular apoptosis more than 2 times more intensively than in the case of control cells, in samples without nanoparticles. Control cells themselves showed low mortality, at the level of approximately 20%, which remained stable both at 24 and 48 h in culture (Fig. 7).

#### Cell cycle evaluation

**Fig. 8:**
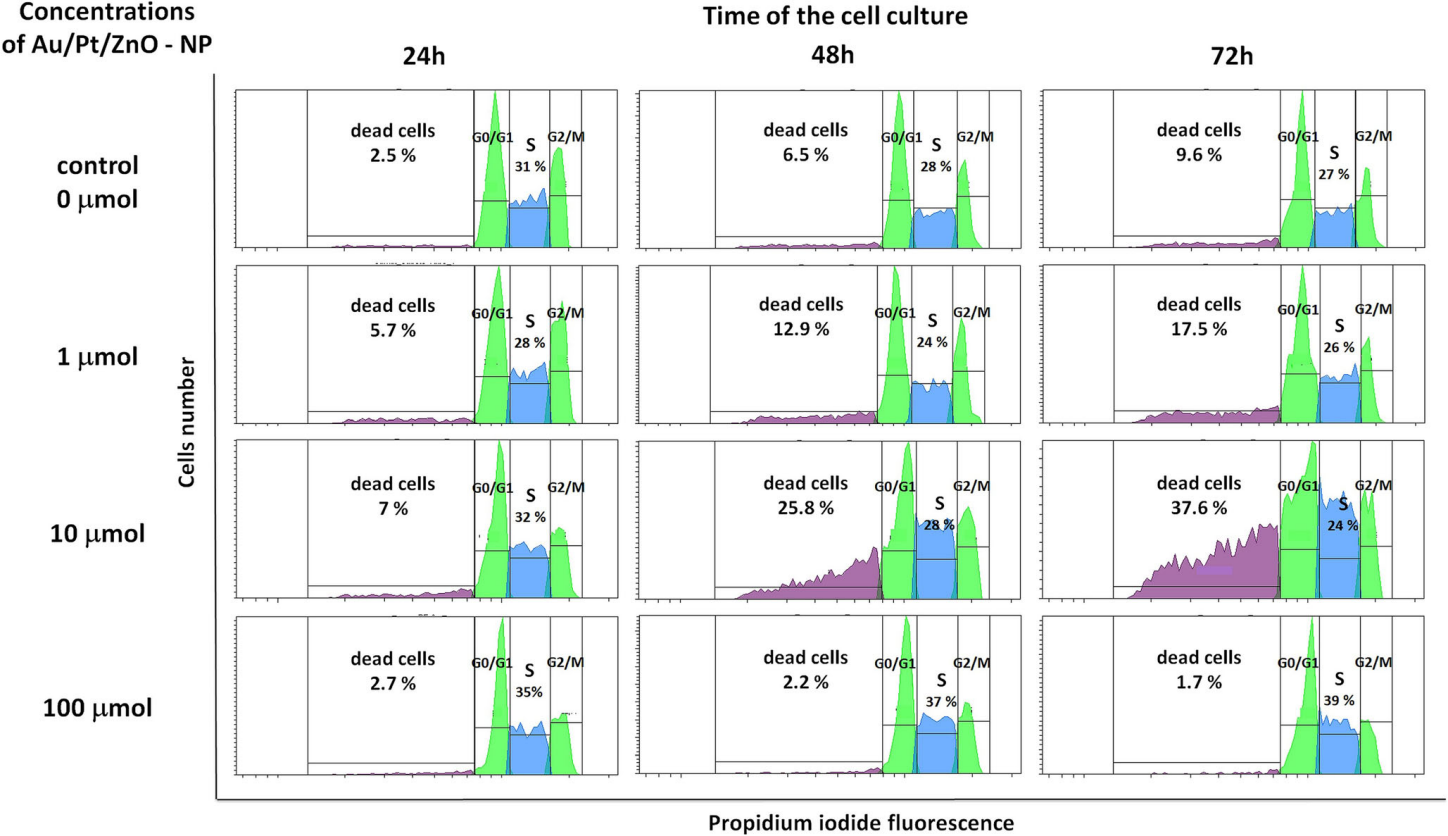
The cell cycle analysis of the Jurkat cell line cultured in the various time period under the influence of various Au/Pt/ZnO nanoparticles concentrations

The cell cycle of Jurkat cells was evaluated in propidium iodide stained cells using a flow cytometer. Test cells were cultured in the presence of 1 μmol, 10 μmol, and 100 μmol of Au/Pt/ZnO nanoparticles for 24, 48 and 72 h (Fig. 8).

**Fig. 9:**
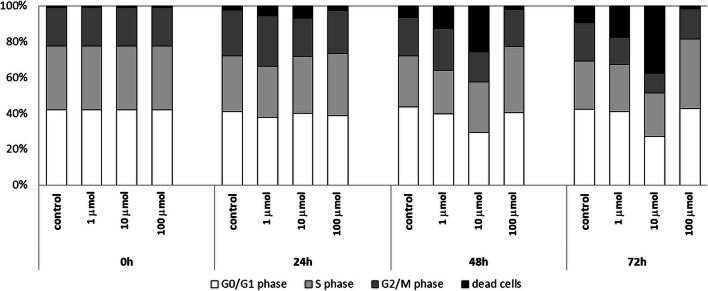
Percentages of Jurkat cells cultured for 24, 48, and 72 h in the presence of different concentrations of Au/Pt/ZnO nanoparticles remaining in G01/G1, S and G2/M phases of the cell cycle

The percentage of dead cells increased with the duration of cell culture and the concentration of applied Au/Pt/ZnO nanoparticles. As in the MTT assay and the Annexin V binding test described above, the most intense cell death process was observed under the influence of the tested nanoparticles used at 10 μmol concentration. Under the influence of nanoparticles administered in the highest concentration of 100 μmol, an intensive decrease in the total number of cells possible to be analyzed was observed. Whereas among the cells that could be assessed, an increase in the percentage of cells in the G2/M phase was observed, which suggests the G2/M arrest process resulting in cell death (Fig. 9).

**Fig. 10:**
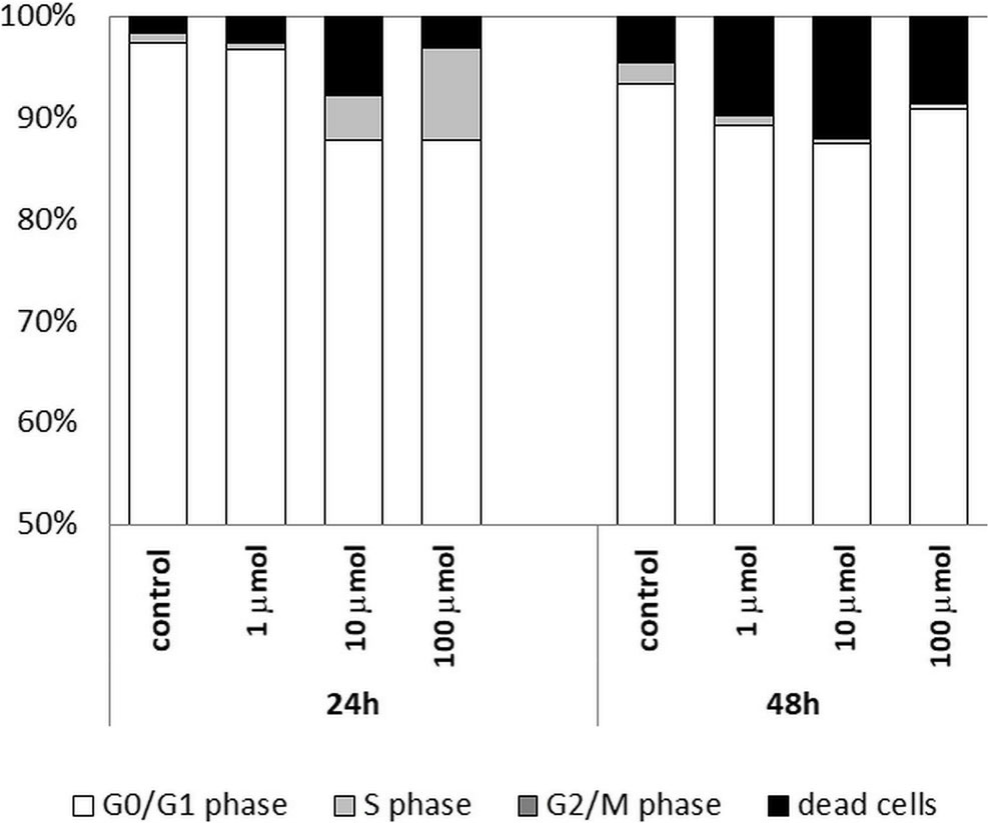
Percentages of peripheral blood lymphocytes of healthy donors cultured for 24, and 48 h in the presence of different concentrations of Au/Pt/ZnO nanoparticles remaining in G01/G1, S and G2/M phases of the cell cycle

During the assessment of PBMCs isolated from whole peripheral blood, the focus was on the cell cycle analysis of peripheral blood lymphocytes. As with Jurkat cells, a significant increase in dead cells was observed relative to samples grown without nanoparticles. The percentage of dead cells increased simultaneously with the increase in the concentration of Au/Pt/ZnO nanoparticles and the duration of cell culture (Fig. 10).

**Fig. 11:**
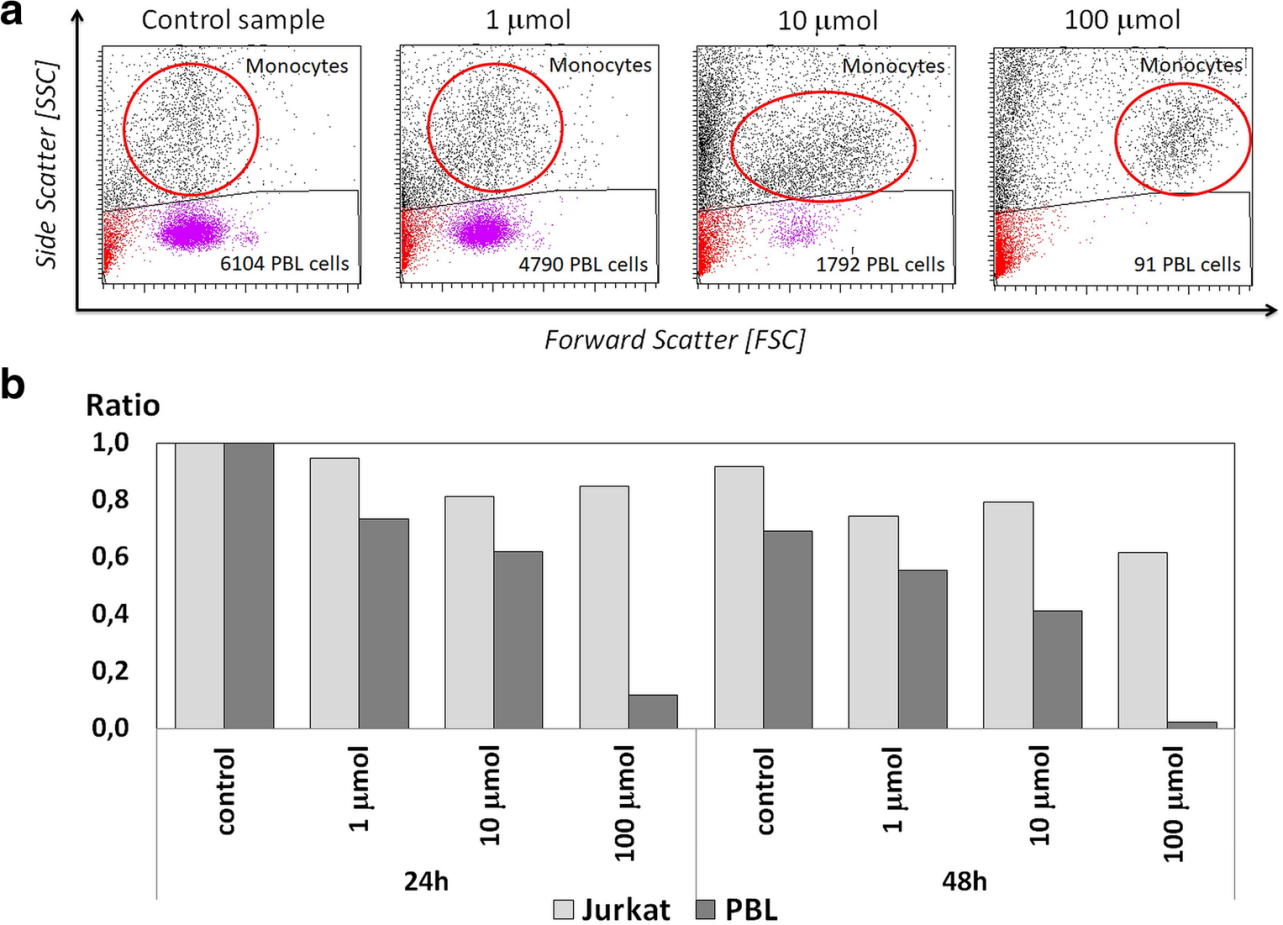
A decreasing number of peripheral blood lymphocytes (PBLs) and increasing monocyte size, accompanying the various concentrations of Au/Pt/ZnO nanoparticles (**A**); Changes in the number of cells during culture in the presence of Au/Pt/ZnO nanoparticles at various concentrations through 24 and 48 h presented in the form of a ratio between the number of Jurkat cells and the number of PBLs isolated from healthy donors (**B**)

Based on the calculated ratio of the number of cells in test samples cultured with Au/Pt/ZnO nanoparticles in various concentrations to the number of cells in the control sample, without nanoparticles, it was found that Au/Pt/ZnO nanoparticles dramatically reduced the number of viable PBL cells, in proportion to the concentration and cell culture time. At the same time, as noted on the basis of the FSC parameter, the volumes of peripheral blood monocytes present among PBMCs were distinctively increased. FSC, or forward scatter, is a parameter proportional to the size of the cell (Fig. 11).

Due to high absorption in NIR and visible range as well as effective production of thermal energy, metal nanoparticles are becoming a promising weapon against cancer. Also, some nanoparticles have been utilized for biochemical modulation and, in the future, could be applied in clinical cancer chemotherapy (Guo et al. 2008). Many scientific works report on the cytotoxic properties of mono-nanoparticles of silver, zinc oxide (Dobrucka et al. 2018), gold (Dobrucka et al. 2019) and others. In this work, Au/Pt/ZnO trimetallic nanoparticles biosynthesized using *A. lappa* extract were used against leukemia, a type of blood or bone marrow cancer, which is still one of the most common and aggressive cancers (Shan et al. 2014). In addition, conventional drugs are not effective in penetrating into the spinal cord or brain. Leukemia cells flourish in these central nervous system hideouts, eventually causing fatal complications (Soni & Yadav 2015). Therefore, scientists are looking for new solutions to treat this kind of cancer. One example of applying metal nanoparticles in leukemia treatment is provided by the studies of Saravanakumar et al. (Saravanakumar et al. 2018), who demonstrated that zinc containing chitosan nanoparticles were successfully generated for treating human acute T lymphocyte leukemia. The combination of zinc and chitosan nanoparticles effectively induced apoptosis in 6 T-CEM cells through the activation of the FAS/CD95, pro-apoptosis and defense-related gene expressions. The studies carried out by Guo et al. (Guo et al. 2008) showed that different sized ZnO nanoparticles exposed to leukemia K562 and K562/A02 cancer cells could exert dose-dependent cytotoxicity suppression in vitro, illustrating the apparently enhanced cellular uptake of daunorubicin with the aid of different sized ZnO nanoparticles on target cancer cells. Guo et al. (Guo et al. 2013) showed that PVP-coated silver nanoparticles of various sizes had anti-leukemia effects on multiple human AML cell lines and primary isolates from AML patients.

## Conclusion

Nanotechnology brings suitable resources by providing nanoparticles with small molecular weights to improve the efficiency of therapy. The synthesis of metal nanoparticles is an enormous and growing field, as they may be applied in various areas, including medicine and pharmacy. One example is biological synthesis, which has gained considerable attention in the recent years due to its numerous advantages, including low costs and the lack of necessity to use toxic solutions. In this work, Au/Pt/ZnO nanoparticles were obtained from *A. lappa* extract. The assessment of the optical properties, size and shape of Au/Pt/ZnO nanoparticles was carried out by means of the following methods: UV-vis, FTIR, SEM, TEM and AFM. The applied methods demonstrated that the size of the obtained spherical nanoparticles ranged from 10 to 40 nm. FTIR confirmed the presence of biologically active compounds in *A. lappa* extract. Our studies also demonstrated that the percentage of dead leukemia cells increased with cell cultivation time and the concentration of Au/Pt/ZnO nanoparticles. The cytotoxicity of Au/Pt/ZnO nanoparticles determined by means of the MTT test indicated that the viability of leukemia cells practically disappeared when the concentration of the tested nanoparticles was 10 mol. The obtained results are the basis for further research to develop new, clinically significant therapeutic agents and biomaterials in research on leukemia and other types of cancer.
